# The impact of severe plastic deformations obtained by hydrostatic extrusion on the machinability of ultrafine-grained Ti grade 2 intended for fasteners

**DOI:** 10.1038/s41598-022-20339-9

**Published:** 2022-09-28

**Authors:** Jacek Skiba, Mariusz Kulczyk, Sylwia Przybysz-Gloc, Monika Skorupska, Krzysztof Niczyporuk

**Affiliations:** 1 Institute of High Pressure Physics, Polish Academy of Sciences (Unipress), ul. Sokołowska 29/37, 01-142 Warszawa, Poland; 2 Center of Innovation and Expertise, Association of Polish Engineers and Mechanical Technicians, Warsaw Department, ul. Czackiego 3/5, 00-043 Warszawa, Poland

**Keywords:** Mechanical engineering, Metals and alloys, Design, synthesis and processing

## Abstract

The study aimed to examine the effect of the hydrostatic extrusion (HE) process on the machinability of Ti grade 2 (Ti) in the turning process. After the deformation with true strain ɛ = 2.28, the microstructure was significantly refined to a grain size of 100 nm, resulting in an increase in the mechanical properties, *UTS* strength by 190%, *YS* yield strength by 230%Cutting forces for Ti in the initial state and after HE were analyzed at cutting depths *a*_*p*_ = 0.3 mm and 0.5 mm, the variables were cutting speed *V*_*c*_ (20, 30 and 50 m/min) and feed rate *f* (0.08 and 0.13 m/s).The impact of the microstructure refinement in Ti after HE on the high cutting depth machinability deterioration (*a*_*p*_ = 0.7 mm) was identified. This phenomenon is particularly noticeable at lower cutting speeds *V*_*c*_ = 20 and 30 m/min at which cutting forces are higher. Application tests of Ti after HE showed a significantly lower susceptibility to buckling during threading. As a result of the tests carried out for the Ti in the initial state, it was not possible to achieve the tolerance of pitch diameter of the thread required by standards, *d*_*2*_ at two of the three cutting depths tested. In turn, for the Ti after HE, the thread tolerances required by the standards were achieved for all tested cutting depths.

## Introduction

The rapid development of technology is associated with the need to search for new structural materials that meet increasingly stringent requirements and allow further technological barriers to be overcome, which until a few years ago were beyond the reach of designers. Therefore, in material engineering, unusual manufacturing methods involving the use of severe plastic deformation (SPD) are increasingly used. However, due to their technological conditions, most SPD methods remain in the domain of scientific research, and only a few have found their way to industrial application.

One of the SPD methods providing high structural homogeneity of deformed materials while at the same time very high process efficiency is hydrostatic extrusion HE. This technology has been developed for many years at the Institute of High-Pressure Physics of the Polish Academy of Sciences (IHPP PAS) and is used to analyze the susceptibility of materials to severe plastic deformation. Long-term studies carried out using high hydrostatic pressures have confirmed the uniqueness of the HE method in giving deformed materials properties unattainable using conventional manufacturing methods such as conventional extrusion, drawing, or rolling. The microstructure refinement obtained in the materials studied enabled them to be given completely new mechanical properties^1–4^, physical properties^5–7^, and performance properties^8–11^.

As the application of SPD processes is increasingly being reported, the need becomes obvious to develop new technologies to optimize existing processes for forming finished products made of these materials. For many years, the most commonly used forming process has been machining, which is required to become even more productive, efficient, quality-assuring, and reliable. Meeting these requirements for a completely new group of materials such as those after SPD processes is extremely difficult. In addition, the matter is complicated by the fact that most of these materials, due to the structure and mechanical properties modified in the deformation process, require a completely different technological approach, which at best involves changing and optimizing the machining parameters, and sometimes requires other tools.

An example of a material that is considered to be barely machinable, and covered by this study, is titanium Ti grade 2. The main problems associated with titanium machining include a high cutting temperature and quick wear of cutting tools. These difficulties are mainly due to titanium’s properties, such as low thermal conductivity and low modulus of elasticity. These properties have a direct impact on the thermoplastic instability characteristic of titanium during machining and the effect of build-up on the cutting surface of tools, which shortens their service life^12,13^.

Titanium and its alloys due to their properties i.e. first of all, the high strength-to-density ratio, corrosion resistance, and biocompatibility find their application in many industries, especially aerospace, automotive, and medical^14–18^.

In the present study, the authors attempted to analyze the effect of microstructure refinement of Ti grade 2 titanium obtained by hydrostatic extrusion on machinability in the turning process. The change in basic mechanical properties such as strength and yield strength has a direct impact on technological processes, including machining processes. Materials with higher parameters are characterized by greater stiffness and dimensional stability during machining. However, an unconventional deformation method such as the HE process generates a significant number of structural defects in the material, which effectively limit the transport of free electrons and phonons, which consequently reduces thermal conductivity^8^. This fact directly affects both the surface quality of the manufactured products and their dimensional tolerance.

The purpose of the analysis of strength properties as a function of strain carried out in this publication is to optimize the parameters of titanium machining with regard to the application process of turning finished products in the form of fasteners while taking into account strict geometry and shape standards.

## Methodology and scope of the tests

The subject of the tests was Ti grade 2 titanium with the following chemical composition, Table 1.

**Table 1:** Chemical composition of Ti grade 2.

Fe	C	N	H	O	Ti
0.17	0.018	0.009	0.001	0.15	99.59

The basic strength properties determined based on static tensile test and hardness measurements are listed in Table 2.

**Table 2:** Mechanical properties of Ti grade 2 at initial state undeformed).

Notation	Ultimate tensile strength *UTS* (MPa)	Yield strength *YS* (MPa)	Elongation to fracture *ɛ*_*f*_ (%)	Hardness *HV0.2*
Ti grade 2	480	345	27	160

The baseline grain size was *d*_*eq*_ ~ 30 µm, where *d*_*eq*_ is the equivalent diameter defined as the diameter of a circle with an area equal to that of the grain.

HE process parameters were optimized, including unit true strain *ɛ*, plastic deformation rate $\dot{\varepsilon }$, tool geometry i.e. die angle *2α*, and the way the material is lubricated. The HE hydrostatic extrusion process was carried out on presses designed and manufactured by IHPP PAS Unipress, with working pressures up to 1800 MPa, and equipped with a system for cooling the extruded product with running cold water to reduce the adiabatic heating effect. To increase the accumulated plastic deformation, the material was extruded in two stages (cumulatively). Titanium Ti grade 2 was hydrostatically extruded through a conical converging die with die angle of *2α* = 45° from the initial diameter of 50 mm to the final diameter of 16 mm with accumulated true strain *ɛ*_*cum*_ = 2.28.

**Figure 1:**
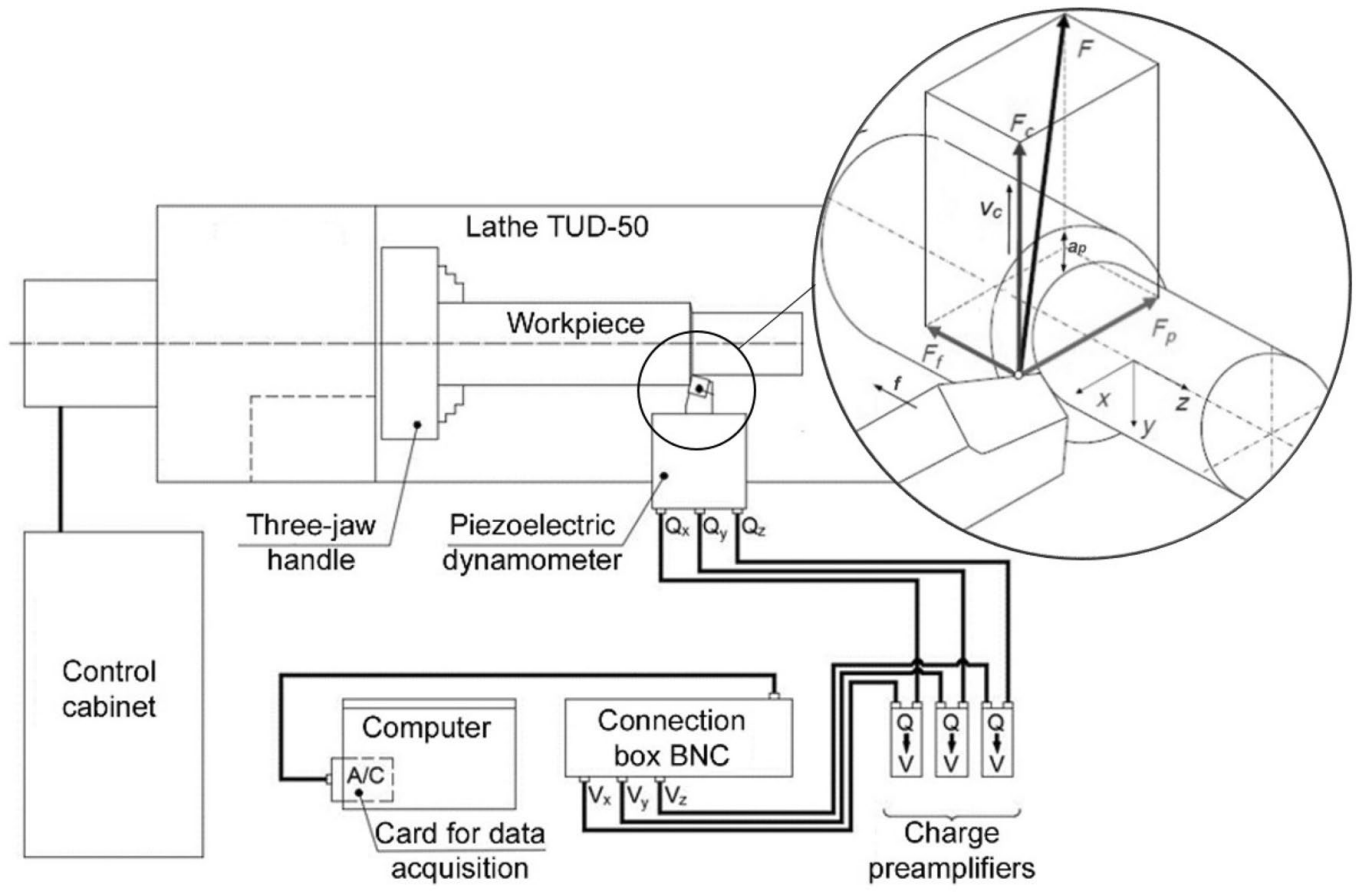
Scheme of the machinability test system.

The initial microstructures were examined with a Nikon Eclipse LV150 LM light microscope, and the microstructures after HE with a TEM JEOL 1200 EX transmission electron microscope. In both cases, cross-sections of an extruded round rods were examined. The grain sizes were quantified with "Mikrometr" software^19^. The data were based on TEM images, where, after the imaging and mapping of at least 200 grains selected at random from the population, the equivalent diameter *d*_*eq*_ was calculated. Mechanical properties were tested using a Zwick-Roell Z250 strength test machine with a maximum force of 250 kN to determine the tensile strength *UTS*, yield strength *YS* and elongation to break *ε*_*f*_*.* The tests were carried out at a tensile strain rate of 0.008 s^−1^ on five-fold samples with a diameter of 6 mm, sampled along the rod axis. Micro-hardness was measured on cross-sections of the extruded rods using a Zwick-Roell hardness tester ZHV1-A under a load of 200 g for 15 s. Component cutting forces were analyzed at a dedicated measuring station provided with a TUD-50 lathe with a Kistler 9623 piezoelectric force sensor that can measure each cutting force: cutting force *F*_*c,*_ feed force *F*_*f*_*,* and passive force *F*_*p*_ with an accuracy of ± 0.01 N, Fig. 1.

The tests were carried out using a tool consisting of an STGCR 1616h11-B1 holder, with a TCGX 110302-AL H10 plate with the following parameters mounted on it:

- Cutting edge angle: *k*_r_ = 91°
- Orthogonal working angle: *g*_*o*_ = 0°
- Cutting edge inclination angle: *l*_*s*_ = 0°
- Plate rounding radius: *r*_*e*_ = 0.2 mm
- Plate material—H10: sintered carbon, uncoated, fine-grained, tungsten carbide-based

Component cutting forces, Fig. 1, were analyzed at a constant cutting depth *a*_*p*_ = 0.3 mm and 0.5 mm, while the variables were cutting speed *V*_*c*_ and feed rate *f*. The cutting speeds for the materials in the initial state and after the HE process were 20, 30, and 50 m/min, and the feed rates were 0.08 and 0.13 m/s.

To verify the component cutting forces, variance analysis of the obtained results was carried out. This analysis included the determination of Pearson's linear correlation *r*_*xy*_ specifying the level of the linear relationship between the variables analyzed and the associated coefficient of determination *R*^2^ which determines the quality of individual components' match to the whole model^20^. Pearson correlation coefficient *r*_*xy*_ varies from -1 to 1, where 1 is an exact positive relationship, and -1 is an exact negative relationship, i.e., if the variable x increases, the variable y decreases, and vice versa. In turn, the coefficient of determination *R*^2^ varies from 0 to 1, and the match improves while *R*^2^ approaches 1.

Surface roughness after machining was measured with a Hommelwerke's Hommel Tester T8000 contact profilometer with a Hommel Etamic TKU300/600 contact head. The selected surface topography parameter *R*_*a*_ was measured under the following conditions: cut-off length *lt* = 4.8 mm, head travel rate *v*_*t*_ = 0.5 mm / s.

## Results and discussion

### Hydrostatic extrusion

The HE process parameters are listed in Table 3.

**Table 3:** Basic parameters of the cold cumulative hydrostatic extrusion HE of Ti grade 2.

Notation/HE pass	Billet diameter *d*_*0*_ (mm)	Product diameter *d*_*f*_ (mm)	True Strain in one pass *ɛ* = lnR^(a)^	Total true strain (cumulative) *ɛ*_*cum*_	*T*_*adiabatic*_ (^o^C)	*T/T*_*m*_^*(b)*^	Extrusion pressure *p*_*HE*_ (MPa)	Linear extrusion speed *V*_*HE*_ (mm/s)	Plastic deformation speed *ἑ*_*HE*_ (s^−1^)
Ti gr2/1	50.00	24.75	1.40	1.40	423	0.36	1101	54.35	3.64
Ti gr2/2	24.75	15.93	0.88	2.28	342	0.32	891	48.55	5.05

^a^R—reduction ratio = initial to final cross section.

^b^T_m_—melting point = 1665 °C.

**Figure 2:**
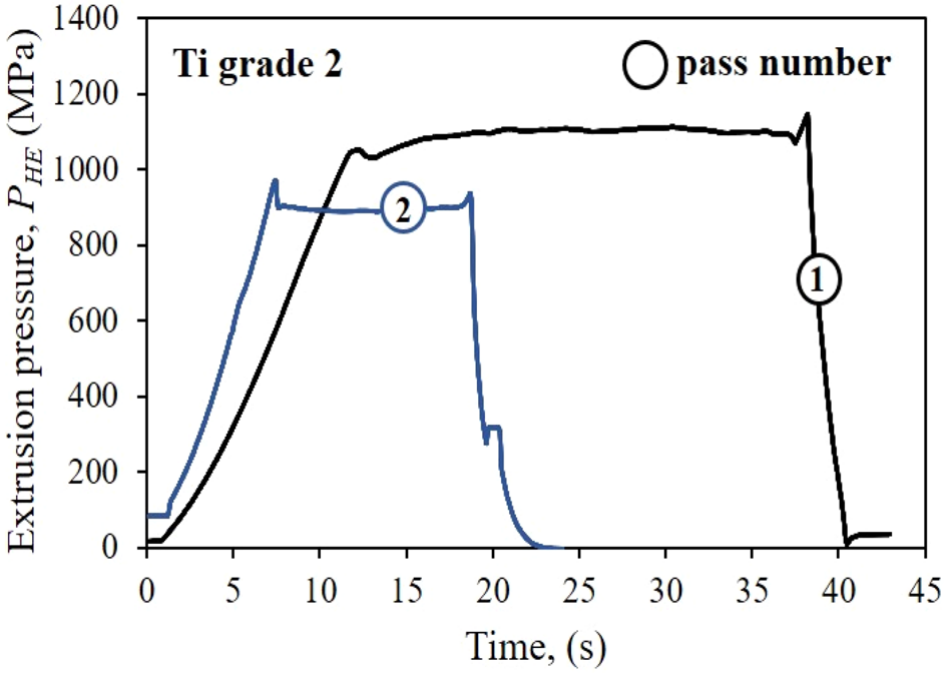
Pressure characteristic of the cold cumulative hydrostatic extrusion of Ti grade 2.

Each stage of the HE process showed a stable extrusion characteristic in the form of "flattening", and the extrusion pressures, in the 1st and 2nd stages respectively, were 1101 MPa and 891 MPa, Fig. 2. In addition, the adiabatic heating effect was analyzed for the HE process. Titanium is not highly susceptible to heat-induced healing and recrystallization processes, however, due to the severe plastic deformation accumulated in the material after the HE process resulting from the increase in the working zone temperature, phenomena such as defect polygonization can occur, which also affects the material's final properties. Such effects were confirmed for Ti grade 2 in the doctoral dissertation ”Study of the effect of severe plastic deformations on thermophysical properties of selected metals”^21^ and in the publication “Effect of severe plastic deformation realized by hydrostatic extrusion on heat transfer in CP Ti grade 2 and 316L austenitic stainless steel”^22^. These studies report thermal effects in the form of healing processes in the titanium after HE with true strain ɛ = 1.16 already at 280 °C, i.e., almost 70% less than in the non-deformed material (ca. 500 °C). The calculated adiabatic heating effect on grade 2 titanium after HE, the results of which are presented here, was for stages 1 and 2 respectively, *T/T*_*m*_ ~ 0.36 and 0.32, where *T* is the temperature measured during extrusion; and *T*_*m*_ = 1665 °C material melting point, both in °C, Table 3.

### Microstructure

**Figure 3:**
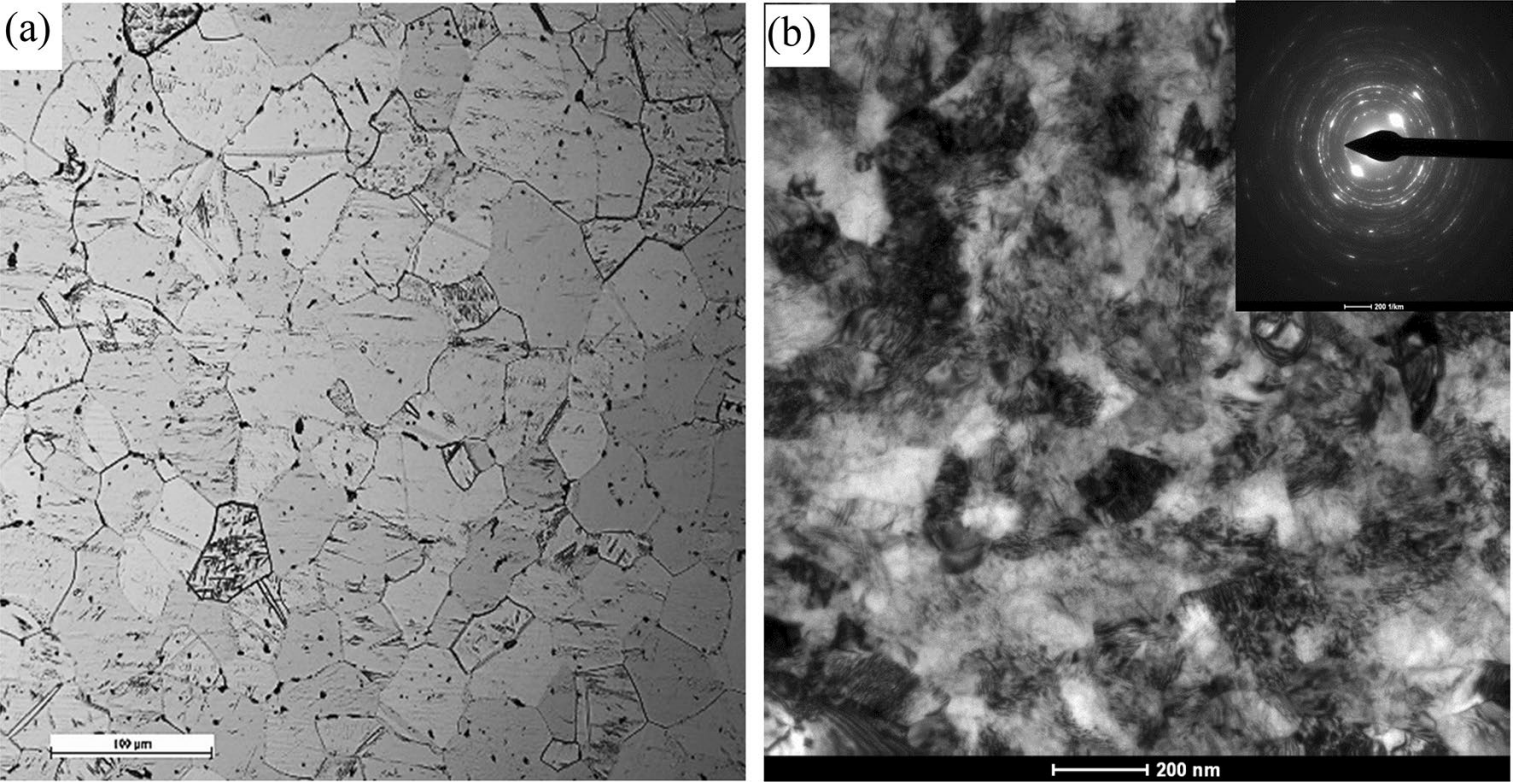
Ti grade 2 microstructure on the cross-section of the rod (**a**) in the initial state, (**b**) after the cold cumulative hydrostatic extrusion process with ε_cum_ = 2.28.

Grade 2 titanium in the initial state had a coarse-grained microstructure with an average grain size *d*_*eq*_ ~ 30 µm, Fig. 3a.

**Figure 4:**
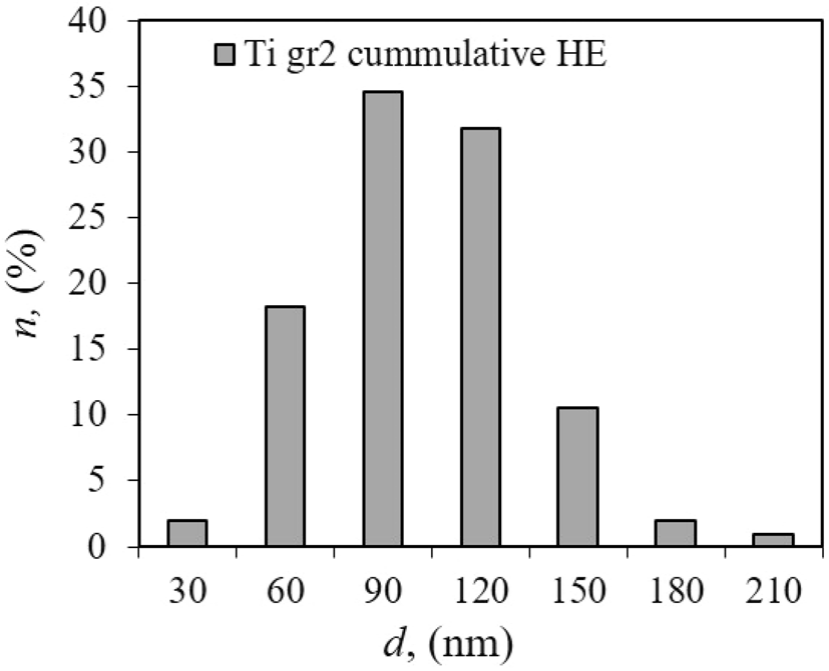
Grain size distribution for Ti grade 2 after the cumulative cold hydrostatic extrusion process with cumulative true strains *ε*_*cum*_ = 2.28.

A two-stage hydrostatic cold extrusion process with cumulative true strain *ɛ*_*cum*_ = 2.28 significantly refined the microstructure as seen in the transmission electron microscope TEM. The average grain size after the cumulative hydrostatic extrusion process is *d*_*eq*_ ~ 100 nm, i.e., nearly 3 orders of magnitude less than in the initial material Fig. 3b. The character of the SAED patterns indicates the majority of grain boundaries are high-angle. This is also evidenced by the grain size distribution typical for well-developed structures, Fig. 4.

### Mechanical properties

**Figure 5:**
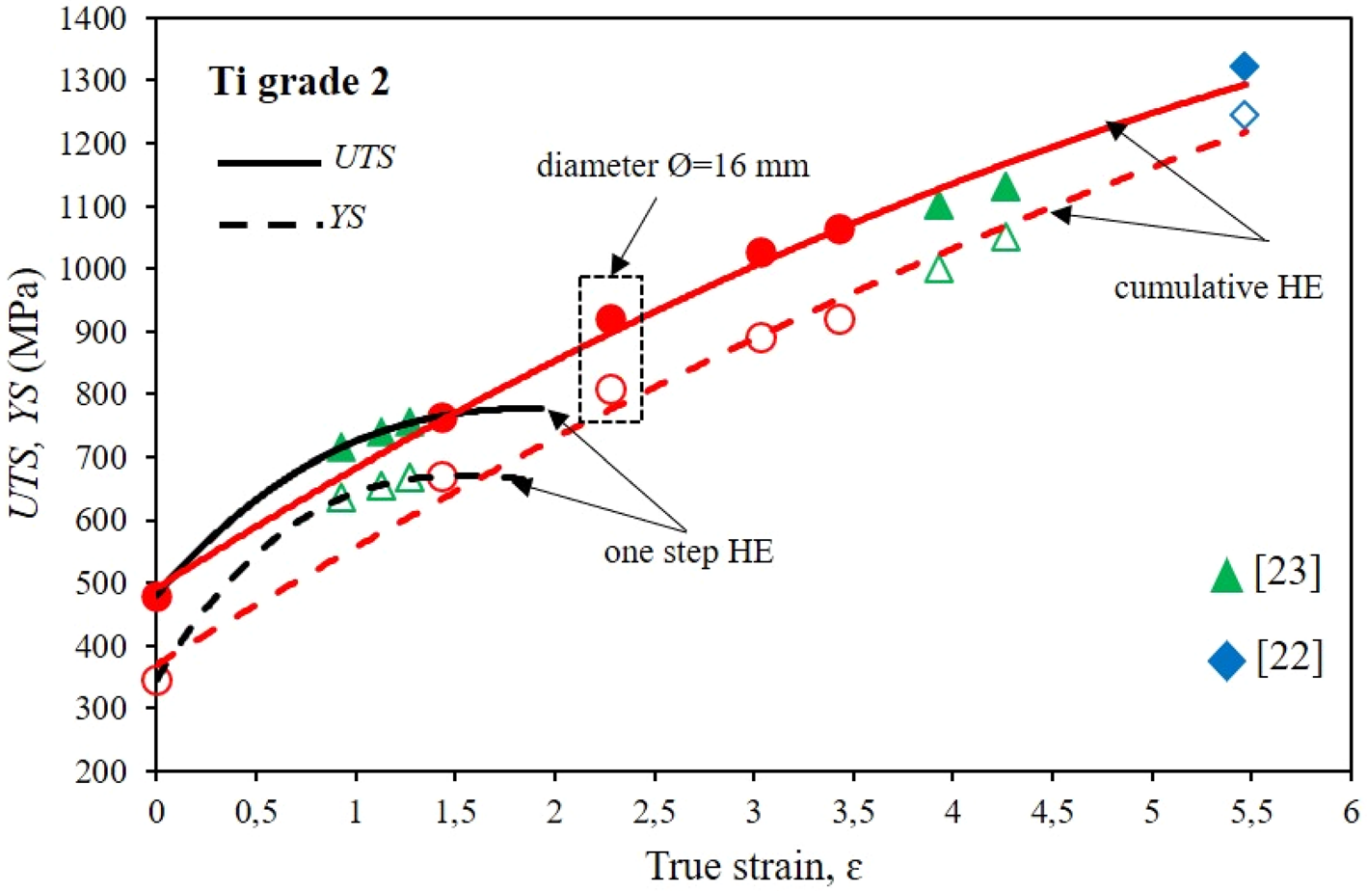
Dependence of *UTS* tensile strength and *YS* yield stress on true strain *ε* for Ti grade 2 after the one-step cold hydrostatic extrusions, and after the cumulative HE.

Figure 5 shows the effects of severe plastic deformation on strength *UTS* and yield strength *YS* of grade 2 titanium after the hydrostatic extrusion process.

Single-stage extrusion with unit true strain incremental from 0.9 to ca. 1.43 indicates defect saturation increase with incremental deformation and a significant share of adiabatic effects that weaken reinforcement effects. This phenomenon is evidenced by the clear flattening of the characteristic with unit deformation increase seen in the graph, Fig. 5. For various unit deformation degrees, similar *UTS* and *YS* values were obtained in the *UTS* range from 715 to 765 MPa and *YS* range from 635 to 670 MPa. These values represent increases relative to the initial material of ca. 60% in *UTS* and more than 90% in *YS.* The grade 2 titanium tested could be further strengthened with the hydrostatic accumulation extrusion process to a final diameter of ø9 mm. Studies of grade 2 titanium with the use of cumulative hydrostatic extrusion have been repeatedly carried out at the Institute of High-Pressure Physics, however, these studies focused on maximizing the effects of strengthening and the microstructure refinement, which translated into very small end product diameters. An example is titanium hydrostatically extruded in 20 steps with an accumulated true strain of ɛ = 5.47, for which the microstructure was refined to the nanometer level with an average grain size of *d*_*eq*_ = 47 nm^22^. The resulting rods with a diameter of ø3.25 mm significantly limited their application studies. Another example is grade 2 titanium after a 10-step cumulative hydrostatic extrusion process combined with rotary swaging with an accumulated true strain of ɛ = 4.27. As a result of the combination of both processes, rods with a diameter of ø6 mm were obtained, with *UTS* = 1130 MPa and *YS* = 1050 MPA^23^.

The purpose of this work was to analyze grade 2 titanium after the HE process in the form of rods that can be used for fasteners. Therefore, for application tests, the hydrostatic extrusion stage with an accumulated true strain of ɛ = 2.28 and a diameter of ø16 mm, which is optimal from the point of view of the target products, was chosen as shown in Fig. 5. At this deformation stage *UTS* = 920 MPa and *YS* = 810 MPA were obtained, i.e. 190% and 230%, respectively, more than in the initial material.

**Figure 6:**
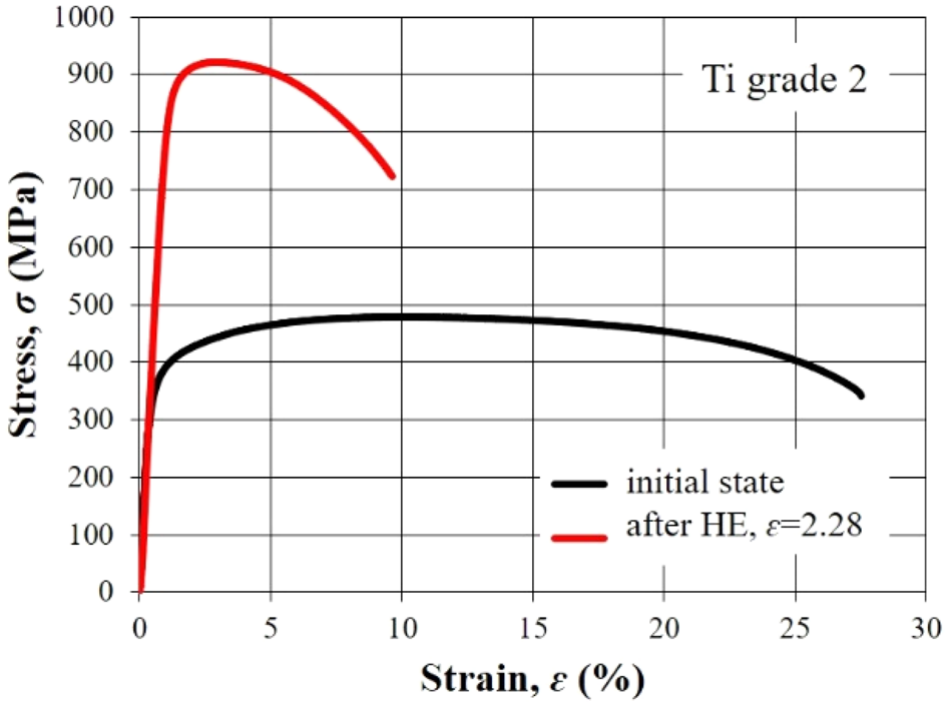
Tensile stress–strain curves for Ti grade 2 in initial state and after hydrostatic extrusion with true train ɛ = 2.28.

Figure 6 shows the static tensile test characteristics for Ti grade 2 in the initial state and after the HE process with true strain *ɛ* = 2.28. The curves obtained reflect clear differences in the structure of the material resulting from strain hardening. It should be noted that despite the very large increase in *UTS* and *YS*, the elongation remains at a relatively high level reaching almost 10% which is about 30% of the value of Ti elongation in the initial state.

**Figure 7:**
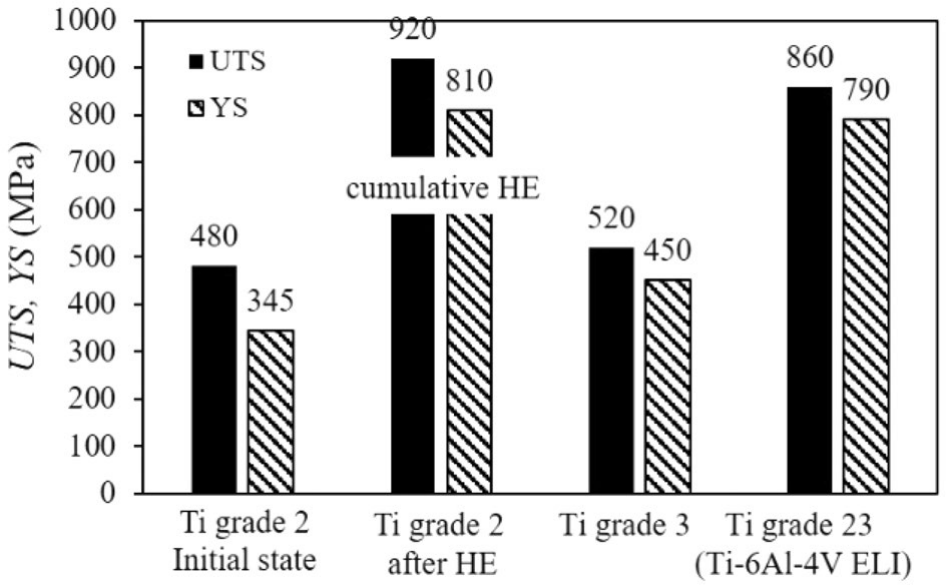
Comparison of *UTS* tensile strength and *YS* yield stress of the Ti grade 2 after the cumulative HE hydrostatic extrusion process with the initial material and commercial alloy requirements for fasteners.

Figure 7 shows a comparison of the mechanical properties of grade 2 titanium in its initial state with the material after the HE process and the most common titanium alloys for industrial applications including fasteners and medical implants^24–28^.

After cumulative HE, the grade 2 titanium has better strength properties than the commercial titanium grades 2 and 3, but also the grade 23 titanium (Ti-6AL-4 V Eli) widely used in medicine. The Al and V admixtures in grade 23 titanium aim to increase the strength to the level required for biomedical applications, which is especially valid in the case of small details in the form of implants. Unfortunately, the alloy admixtures do not remain neutral for the organism, as repeatedly confirmed in publications^29–33^. By generating a severe plastic deformation in pure grade 2 titanium, a higher strength and yield strength were obtained than those of grade 23 titanium alloy. The *UTS* and *YS* values for grade 2 Ti after HE were higher by 7% and 3% respectively, Fig. 7.

### Analysis of cutting forces

**Figure 8:**
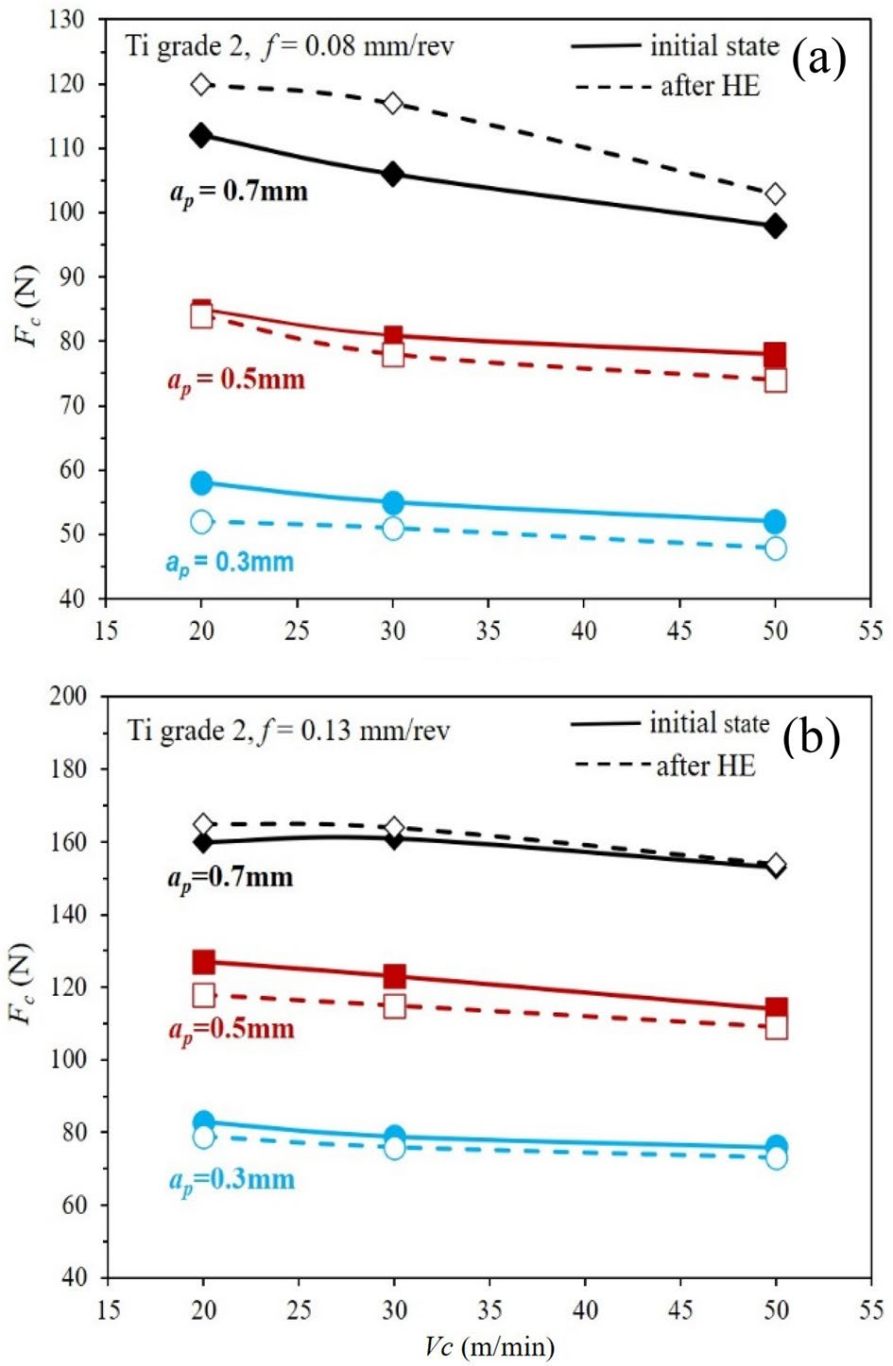
Components of the cutting force, *F*_*c*_, for the feed rate *f* = 0.08 mm/rev (**a**), and *f* = 0.13 mm/rev (**b**) as a function of the cutting speed *V*_*c*_ for Ti grade 2 in the initial state and after the cold cumulative hydrostatic extrusion HE process.

The machinability analysis in the turning process of grade 2 titanium showed changes in the main cutting force component, i.e. *F*_*c*_ in the tested range of variables such as feed rate, *f* and cutting depth, *a*_*p*_ (both as functions of cutting speed, *V*_*c*_), Fig. 8. The other components, i.e., *F*_*f*_ and *F*_*p*_*,* slightly oscillated within the measurement error range. For the titanium after HE, the *F*_*c*_ component decreased, at *f* = 0.08 and 0.13 and *a*_*p*_ = 0.3 and 0.5 over the full range of cutting speed *V*_*c*_ tested, from 20 to 50 m/min relative to the material in the initial state. This effect is associated with strong microstructure refinement of the material and its strengthening, which promotes proper chip formation and reduces build-up on the cutting tool's working surface. This phenomenon has been observed repeatedly in the literature for nanocrystalline titanium, with respect to various machining methods. This is confirmed by research conducted by Lapovok, in which the authors analyzed the machinability of turning Ti grade 2 after the ECAP process^34^. In that publication, the authors concluded that a viable recipe for improving the machinability of titanium is to reduce the grain size below the level of about 500 nm. Another example is the analysis of cutting forces in the Ti grade 2 milling process analyzed by Chabrat^35^. The authors showed that the values of the cutting force component *Ft* during the milling process of nanocrystalline titanium with an average grain size of 110 nm are smaller by as much as 40% compared to its coarse-crystalline form. At the same time, the cutting force models developed by the authors indicate a significant effect of depth of cut and feed rate in the range of cutting parameters analyzed, which is also confirmed in this publication.

The effect of grain size on machinability is also confirmed by studies of machinability in the drilling process carried out by Campos^37^. The microstructure obtained in the 2 × ECAP process with an average grain size of 1.32 µm led to a decrease in machinability and an increase in the principal component by almost 25%. This effect may be related to the critical microstructure refinement of titanium below 500 nm mentioned by Lapovok^34^. The effects of microstructure refinement on machinability were also observed in titanium alloys^38,39^, but also in other materials such as aluminum alloy^40,41^ and copper^42,43^ characterized by a refinement microstructure obtained by plastic deformation processes with severe deformations. After a cutting depth increase to *a*_*p*_ = 0.7 mm, component *F*_*c*_ characteristics as a function of cutting speed were reversed relative to those for lower cutting depths (*a*_*p*_ = 0.3 and 0.5 mm). In this case, the *F*_*c*_ component is higher for the titanium after HE than for the material in the initial state. This effect is evident at both feed rates *f* = 0.08 and *f* = 0.13. Such effects have been reported in the literature by Chabrat, but not discussed. This effect was evidenced for the grade 2 titanium after the HE process at the following machining parameters: *V*_*c*_ = 50 m/min, *a*_*p*_ = 0.4 mm, and F from 0.1 to 0.3^36^. The *F*_*c*_ component increase in the titanium after HE at high cutting depths is probably due to a decrease in the thermal conductivity of titanium because of numerous defects developed in the plastic deformation process. These defects are crucial in the heat exchange process by effectively reducing it. The authors evidenced this phenomenon in the case of studies of Ti grade 2 and 316L steel for medical implants^8^. These studies have shown a decrease in basic thermophysical parameters such as thermal diffusivity and specific heat, which decreased with an increase in plastic deformation by up to twelve or so percent compared to the material in the initial state. This limitation of effective heat exchange in the turning process at high cutting depths of the order of *a*_*p*_ = 0.7 mm and more results in the inability to remove heat from the cutting zone during machining, which consequently leads to build-up and translates directly into an increase in cutting forces. Based on the tests performed, the individual component cutting forces for grade 2 titanium in the initial state and after the HE process were determined using the least-squares method^44^.

Cutting force component *F*_*c*_:

1 $${F}_{c}={C}_{c}\cdot {f}^{{y}_{c}}\cdot {{a}_{p}}^{{x}_{c}}\cdot {{v}_{c}}^{{z}_{c}}$$

Feed force component, *F*_*f*_:

2 $${F}_{f}={C}_{c}\cdot {f}^{{y}_{f}}\cdot {{a}_{p}}^{{x}_{f}}\cdot {{v}_{c}}^{{z}_{f}}$$

Passive force component, *F*_*p*_:

3 $${F}_{p}={C}_{c}\cdot {f}^{{y}_{p}}\cdot {{a}_{p}}^{{x}_{p}}\cdot {{v}_{c}}^{{z}_{p}}$$

where *C*_*c*_ means processing conditions factor, *a*_*p*_ cutting depth (mm), *f* feed rate (mm/obr), *V*_*c*_ cutting speed (m/min), and *y*_*c*_*, x*_*c*_*, z*_*c*_*, y*_*f*_*, x*_*f*_*, z*_*f*_*, y*_*p*_*, x*_*p*_*, z*_*p*_*,* determined experimentally using least-squares method.

The analytical results are listed in Table 4. In addition, they were subjected to statistical verification consisting of the determination of Pearson correlation *r*_*xy*_ and determination *R*^2^ coefficients. The Pearson correlation coefficient is determined by the formula^20^:

**Table 4:** Constants and exponents for model equations of component cutting forces.

Material condition	Cutting forces component	Material constant, *C*_*i*_	Exponent, *x*_*i*_	Exponent, *y*_*i*_	Exponent, *z*_*i*_	Determination coefficient, *R*^2^	Pearson linear correlation, *r*_*xy*_
Initial state	*F*_*c*_	1538.01	0.79	0.81	− 0.10	1.00	1.00
	*F*_*f*_	174.84	0.78	0.24	− 0.24	0.94	0.97
	*F*_*p*_	4.35	− 1.87	− 0.74	− 0.80	0.81	0.90
After HE	*F*_*c*_	1579.01	0.91	0.78	− 0.11	0.99	1.00
	*F*_*f*_	341.07	0.71	0.44	− 0.29	0.94	0.97
	*F*_*p*_	3.33	− 1.35	− 0.20	− 0.23	0.59	0.77

4 $${r}_{xy}=\frac{n\cdot \sum {x}_{i}\cdot {y}_{i}-\sum {x}_{i}\cdot \sum {y}_{i}}{\sqrt{\left[n\cdot \sum {x}_{i}^{2}-{\left(\sum {x}_{i}\right)}^{2}\right]}\cdot \left[n\cdot \sum {y}_{i}^{2}-{\left(\sum {y}_{i}\right)}^{2}\right]}$$

where *n* is the number of points, *x*_*i*_ is the cutting speed component *V*_*c*_ (m / min), and *y*_*i*_ component of cutting force *F*_*c*_*, F*_*f*_*, and F*_*p*_ (N).

The determination coefficient is determined by the formula^20^:

5 $${R}^{2}={r}_{xy}^{2}$$

where *r*_*xy*_ is Pearson's linear correlation coefficient.

The results obtained indicate a close negative correlation (values *r*_*xy*_ range from − 0.77 to − 1.00), which means that as cutting speed *V*_*c*_ increases, the component forces *F*_*c*_, *F*_*f*_ and *F*_*p*_ decrease.

The determination coefficients of not less than 0.8 indicate a very good matching of the determined component cutting forces with the adopted model. The exception is *R*^2^ = 0.59 for titanium after HE's *F*_*p*_, however, this force component is the lowest, and values of several N are essentially negligible.

### Surface roughness

**Figure 9:**
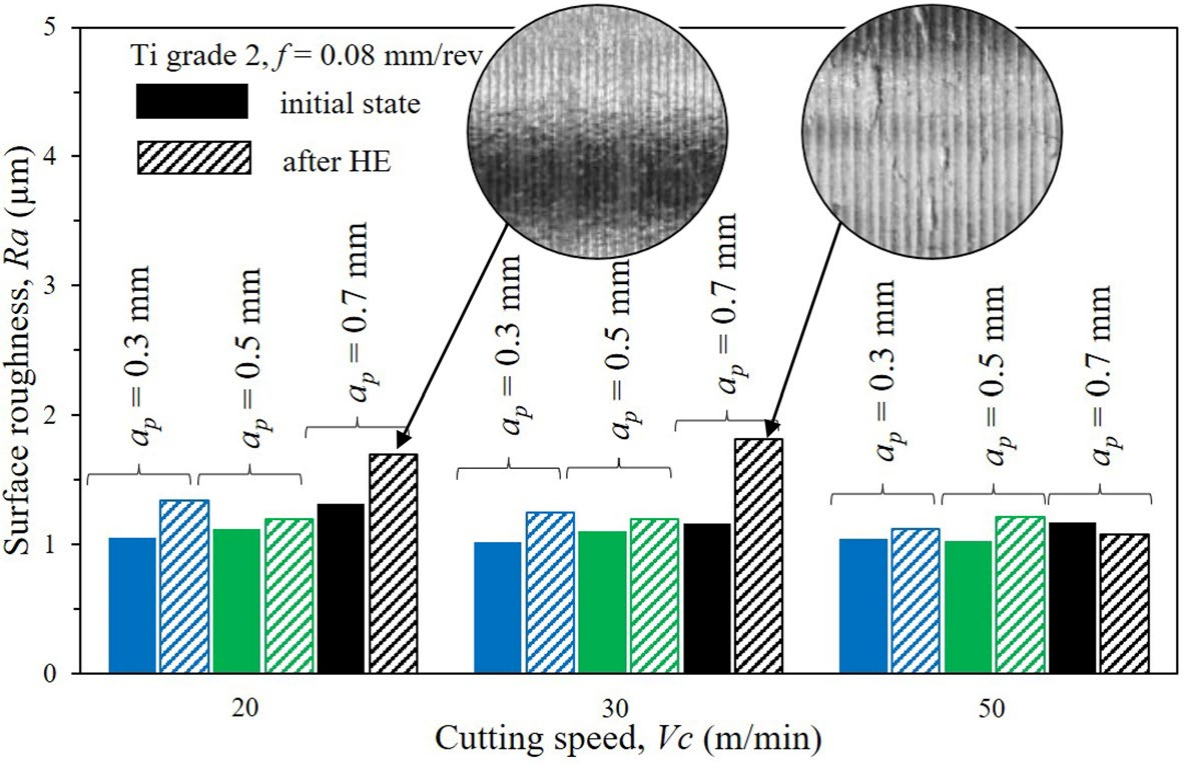
Dependence of the surface quality *R*_*a*_ on the feed rate *f* = 0.08 mm/rev after turning of the Ti grade 2 at the initial state (undeformed) and after the cold cumulative hydrostatic extrusion with the cutting speed *V*_*c*_ = 20, 30 and 50 m/min.

**Figure 10:**
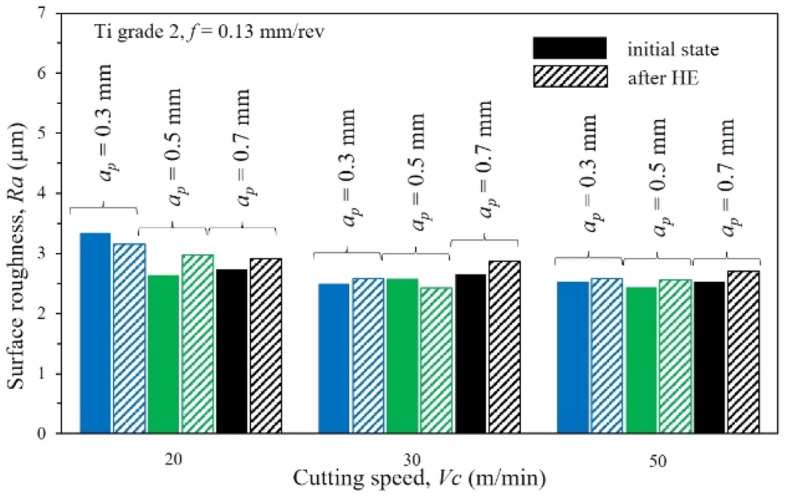
Dependence of the surface quality *R*_*a*_ on the feed rate *f* = 0.13 mm/rev after turning of the Ti grade 2 at the initial state (undeformed) and after the cold cumulative hydrostatic extrusion with the cutting speed *V*_*c*_ = 20, 30 and 50 m/min.

The analysis of surface roughness of the grade 2 titanium machined by turning showed a comparable or slightly worse surface quality of the samples after the hydrostatic extrusion process compared to the material in the initial state, Figs. 9 and 10. Comparable surface roughness is seen in all test samples except two after the HE process machined with a feed rate of 0.08 mm/rev and a cutting depth of 0.7 mm at cutting speeds of 20 and 30 m/min. In these samples, a clear *R*_*a*_ increase was observed compared to the material in the initial state as shown in Fig. 9.

The clear *R*_*a*_ increase is explained by light microscopy images that show surface defects and blurs on the test samples' treated surfaces resulting from build-up on the cutting p late during the machining process. In addition, the phenomenon of build-up at the greatest of the cutting depths studied is confirmed by studies of component cutting forces that show an increase in *F*_*c*_ component force, Fig. 8. This increase is particularly evident in samples with high roughness coefficients *R*_*a*_ resulting from the build-up during machining.

### Thread outline analysis

**Figure 11:**
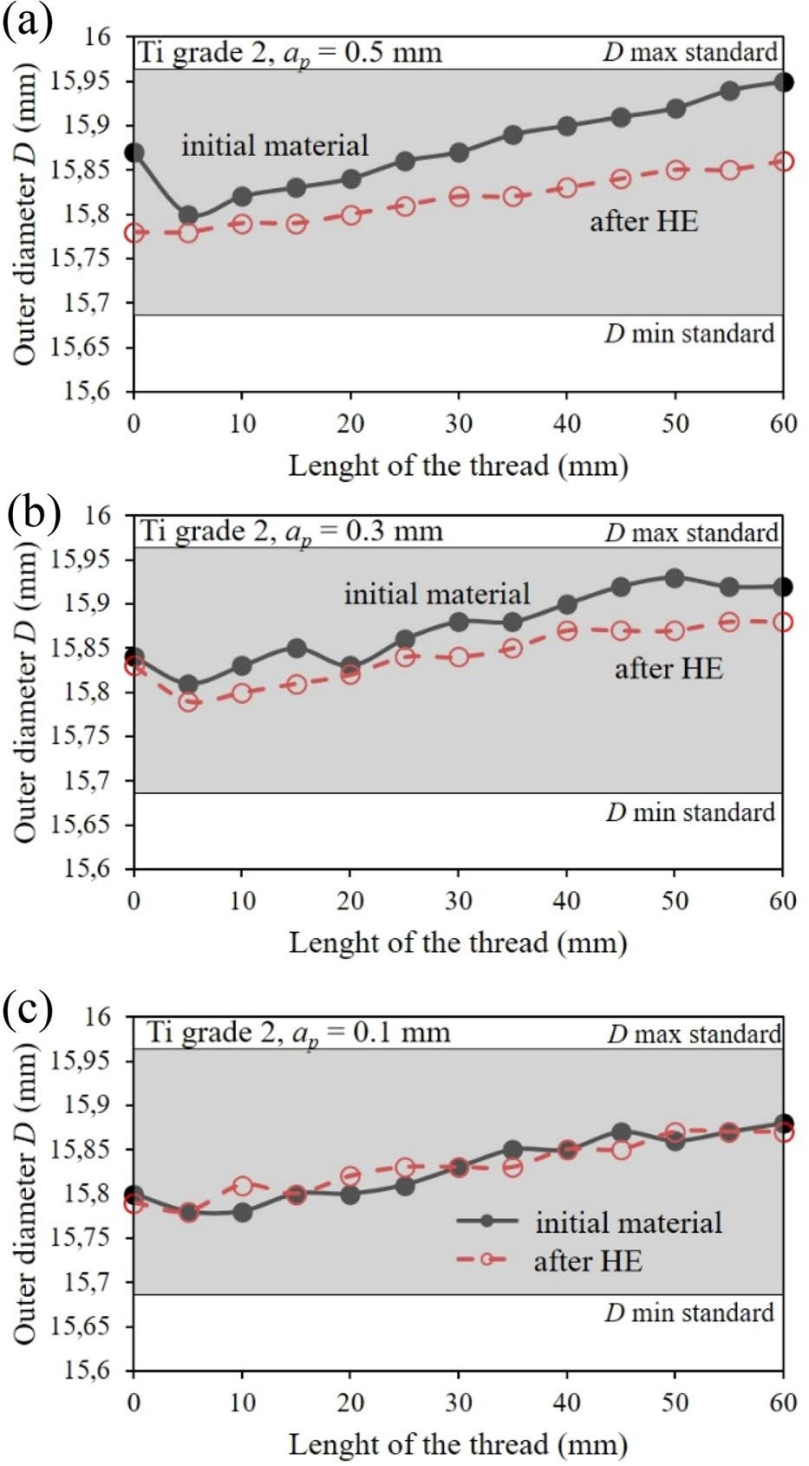
Changes in outer diameter in relation to normalized values over the length of turned threads of commercial Ti grade 2 and after the cold HE hydrostatic extrusion.

**Figure 12:**
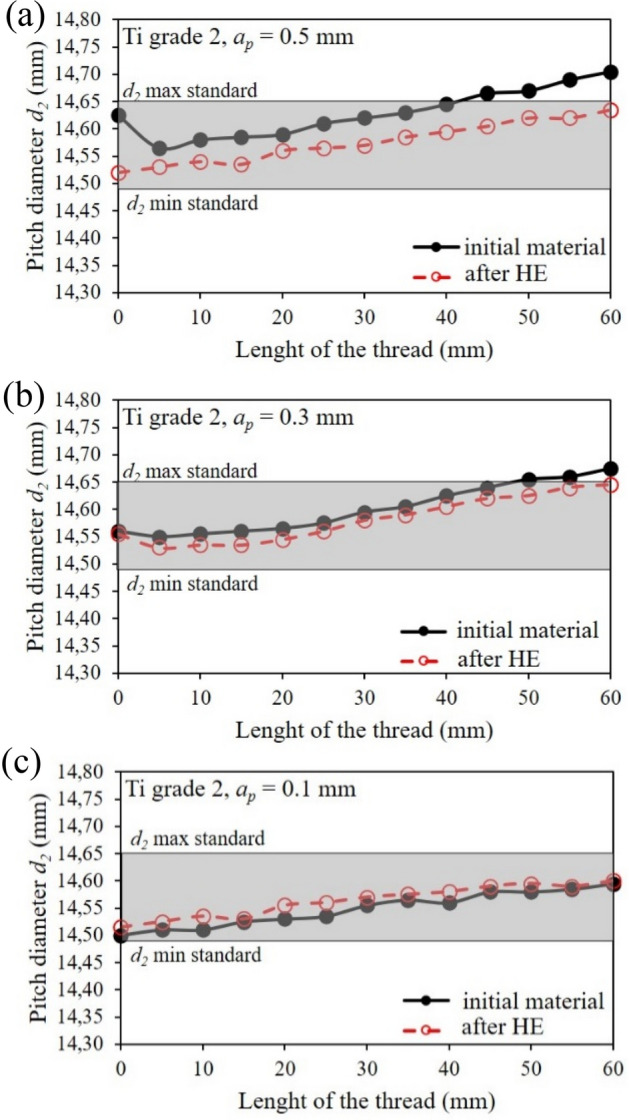
Changes in pitch diameter in relation to normalized values over the length of turned threads of commercial Ti grade 2 and after the cold HE hydrostatic extrusion.

From hydrostatically extruded grade 2 titanium with a diameter of ø16 mm, the fasteners in the form of threaded screws were made by turning according to the tolerance for ordinary external threads in the medium class within the tolerance field 6 g according to the PN-ISO 965-2:2001 Standard. Threads were measured with a Sinpo Profile Projector JT 12A-B measuring microscope, and the measured outline diameter, *D* and pitch diameter, *d*_*2*_ are shown in Figs. 11 and 12. Thread turning tests were performed at cutting depth *a*_*p*_ = 0.3 and 0.5 as analyzed in this publication and for *a*_*p*_ = 0.1 for comparative purposes. The thread turning processes were carried out in each case at a rotational speed *n* = 1000 rpm. Measurements of the outer thread diameter during the tapping process of M16 × 60 mm bolts in the initial state and after the HE process clearly indicated the impact of the hydrostatic extrusion process on machinability in the tapping process. At the greatest cutting depth, i.e., 0.5 mm, the largest differences were observed in the outer diameter measurements of the analyzed threads, which also increased with the thread length, Fig. 11a. This phenomenon is related to the fact that titanium after the HE process has significantly higher strength parameters, which significantly reduces bulging of the machined material. This effect is very clear because the object of the test is a rod with a diameter of ø16 mm, which additionally negatively affects its rigidity. The differences in the outer thread diameter measurements decrease as the cutting depth decreases during the tapping process, and *a*_*p*_ = 0.1 mm the results for the material in the initial state and after the HE process overlap to a large extent, Fig. 11c. In each test variant of the turning process, the outer thread diameter was within the permissible tolerances in accordance with the PN-ISO 965-2:2001 Standard, while at the greatest cutting depth, i.e., *a*_*p*_ = 0.5 mm, at a distance of 60 mm, the outer diameter reaches the maximum permissible value according to PN-ISO 965-2:2001, Fig. 11a.

**Figure 13:**
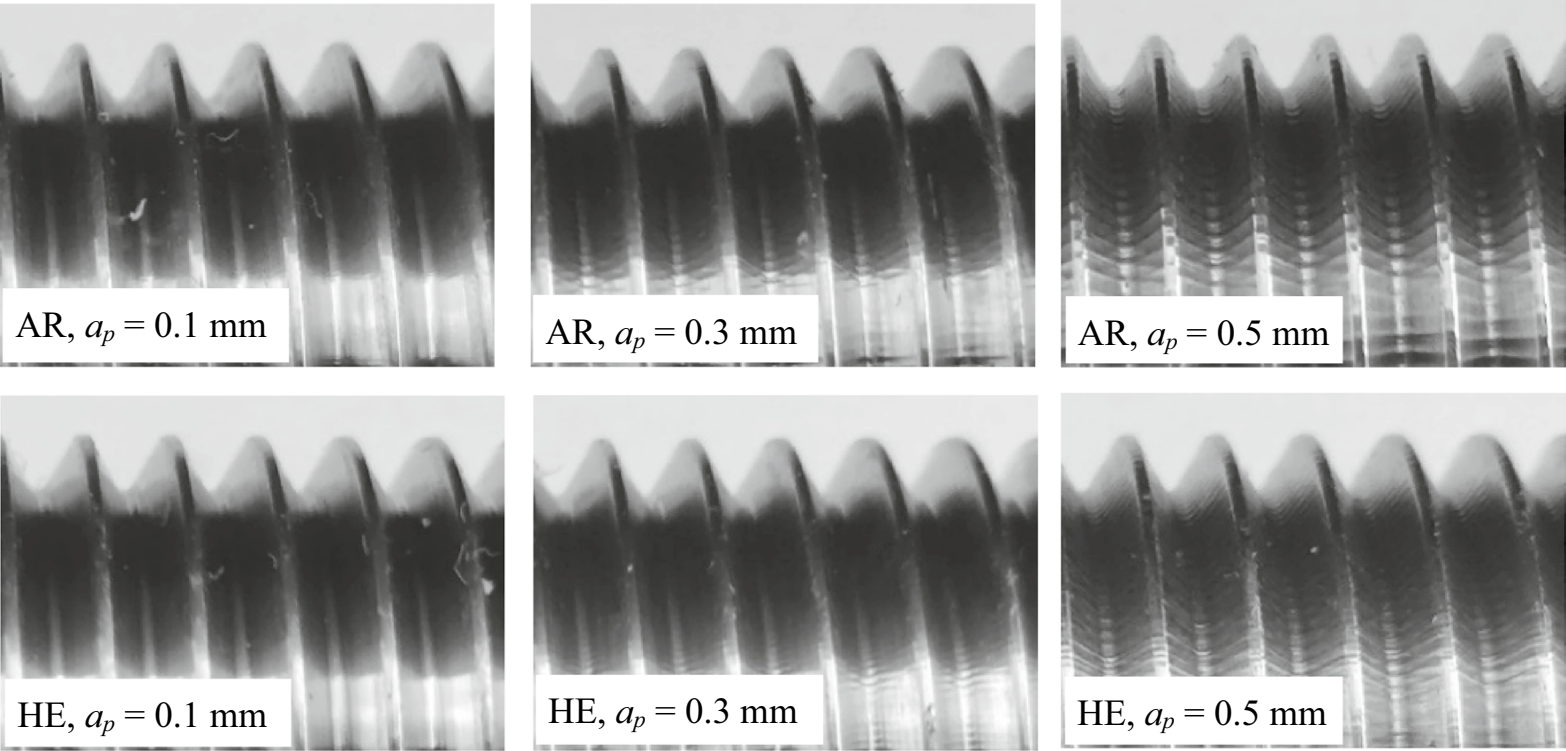
Analysis of the profile of turned threads made of grade 2 titanium in the initial state and after the HE process.

Similarly, the thread pitch-diameter measurements are shown at Fig. 12. As the cutting depth increases, the cutting diameter increases *d*_*2*_. This tendency intensifies further, as in the outer thread diameter measurements (Fig. 11), as the threaded section length extends. This phenomenon is caused by the decrease in the machined rod's stiffness with an increase in the machined section length, i.e., by moving the cutting tool away from the spindle holder. This effect is particularly evident in the case of deeper cutting depths, where the positive effects of the titanium reinforcement after the HE process can be seen, which translates into more rigidity during machining, Fig. 12a,b. In the tapping process with cutting depth *a*_*p*_ = 0.3 and 0.5 mm in the tested thread length range, i.e., 60 mm, the tolerance required for the initial state titanium by PN-ISO 965-2:2001 Standard was not obtained, Fig. 12a,b. In the case of the titanium tapping after HE, in each of the treatment variants tested the thread pitch diameter *d*_*2*_ is within the dimensional tolerance permitted by PN-ISO 965-2:2001 Standard, Fig. 12. The threads were then subjected to macroscopic optical analysis, the results of which are shown in Fig. 13. This analysis showed a significant impact on both processing parameters and material conditions. In both test materials, i.e., titanium in the initial state and after the hydrostatic extrusion process, a deterioration in surface quality was observed with an increase in cutting depth. This phenomenon is associated with the occurrence of vibrations generated during the machining process, which form a characteristic structure on the thread outline surface. This effect is less noticeable in the titanium after the HE process, which is due to the previously described deformation strengthening effects that increase the strength and rigidity of hydrostatically extruded grade 2 titanium rods, Fig. 13.

## Conclusions

The paper examines the impact of severe plastic deformations obtained by hydrostatic extrusion HE on the mechanical and structural properties of grade 2 titanium, which directly affect the susceptibility to machining by turning of commercial products, i.e., fasteners.

The grade 2 Ti obtained after the cumulative HE process with total true strain *ɛ* = 2.28 was characterized by a refined microstructure with medium grain size *d*_*eq*_ = 100 nm which contributed to a significant increase in mechanical properties compared to the initial material.

At this deformation stage *UTS* = 920 MPa and *YS* = 810 MPA were obtained, i.e., 190% and 230%, respectively, more than in the initial material. The above mechanical properties were obtained in rods with a diameter of ø16 mm, which were subjected to a machinability test at a dedicated measuring station.

The machinability analysis in the turning process of grade 2 titanium showed changes in the main cutting force component, i.e., *F*_*c*_ in the tested range of variables such as feed rate f and cutting depth *a*_*p*_ (both as functions of cutting speed *V*_*c*_). The other components, i.e., *F*_*f*_ and *F*_*p*_ slightly oscillated within the measurement error range. For the titanium after HE, the *F*_*c*_ component decreased, at *f* = 0.08 and 0.13 and *a*_*p*_ = 0.3 and 0.5 over the full range of cutting speed *V*_*c*_ tested, from 20 to 50 m/ min relative to the material in the initial state. An increase in cutting depth to *a*_*p*_ = 0.7 contributed to the increase in component *F*_*c*_ above the values*,* achieved for titanium in the initial state. This phenomenon is caused by a significant increase in temperature during the turning process, which is not dissipated by heat exchange. The strong microstructure refinement and accumulation of high-energy defects in the titanium after HE significantly impede heat exchange during the turning process, which results in a build-up of a layer of material on the cutting plate edge. Consequently, this leads to work surface quality deterioration and an increase in individual cutting forces, which is not observed in the non-deformed material. Nevertheless, with properly selected machining parameters that take into account the limited heat exchange of nanocrystal titanium after HE, products with smaller diameters and longer lengths can be manufactured without the risk of non-compliance with strict standards and tolerances for precision fasteners. This is possible by increasing the strength and rigidity of the HE-processed rods, which neither buckle nor deform in the machining process.

## Data Availability

The datasets used and/or analyzed during the current study available from the corresponding author on reasonable request.
